# Books and Magazines

**Published:** 1914-03

**Authors:** 


### Books and Magazines.

Treatment of Rheumatic Infections. From the press of Parke, Davis & Co.

This is a summary of articles on Phylacogens, and Dr. Schafer's discovery, and his views on the treatment of rheumatism. Parke, Davis & Co. will send free a copy on request.

Pyorrhea Alveolaris. By Friedrich Hecker, B. Sc., D. D. S., A. M., M. D., Member Academy of Sciences, St. Louis; Consultant of Bell Memorial Hospital of the School of Medicine, University of Kansas, etc. etc. Illustrated. St. Louis, C. V. Mosby Co., 1913. Price $2.00.

Says the author: "The opinions as to the cause and treatment of pyorrhea are as diverse at the present time as they were many years ago. The most common belief of the dentists is that the disease is a local process." The author does not agree with this opinion, but holds that "the disease is the result of constitutional and exciting causes, which lower the vital resistance of the alveolar process, gums, and peridental memberane." "The body," he says, "is out of harmony, physiologically, and, as a result thereof, manifests itself [sic] in the alveolar process, the gum and peridental membrane." I don't suppose he meant to say that the body manifests itself in the gums, but he said it. Dr. Hecker then proceeds to write a book on the subject along this line.

